# An apparent association between North American moose and a native earthworm

**DOI:** 10.1002/ecy.70146

**Published:** 2025-07-02

**Authors:** Donald F. McAlpine, Mary C. Sollows, Gayathri Sreedharan, Gregory F. M. Jongsma, John Warren Reynolds

**Affiliations:** ^1^ Department of Natural History New Brunswick Museum Saint John New Brunswick Canada; ^2^ Oligochaetology Laboratory Kitchener Ontario Canada

**Keywords:** *Alces alces*, *Bimastos rubidus*, *Dendrodrilus rubidus*, earthworm, ecological association, Lumbricidae, New Brunswick

In once‐glaciated eastern North America, nearly all earthworm species present are lumbricids, introduced since European settlement, or more recent pheretimoid introductions of Asian origin (McAlpine et al., 2022; Reynolds, 2022). Among the 29 earthworm species recorded to date from eastern Canada (McAlpine et al., 2022, 2023; Reynolds, 2022), only two are deemed to be native; *Sparganophilus tamesis* is a limicolus species (Sparganophilidae) with a limited distribution in eastern Canada (Reynolds, 2022), and *Bimastos* (*Dendrodrilus*) *rubidus* (Lumbricidae; Figure 1), as a result of recent taxonomic and nomenclatural revision (Csuzdi et al., 2017), is now recognized to be native to North America. The latter species is a corticole (living under the bark of downed trees) and epigeic (litter inhabiting) and most abundant in hardwood stands. *B. rubidus* has been recorded in a variety of disturbed soils in both the northern and southern hemispheres and as a non‐native is now cosmopolitan in distribution (Reynolds, 2022).

**FIGURE 1:**
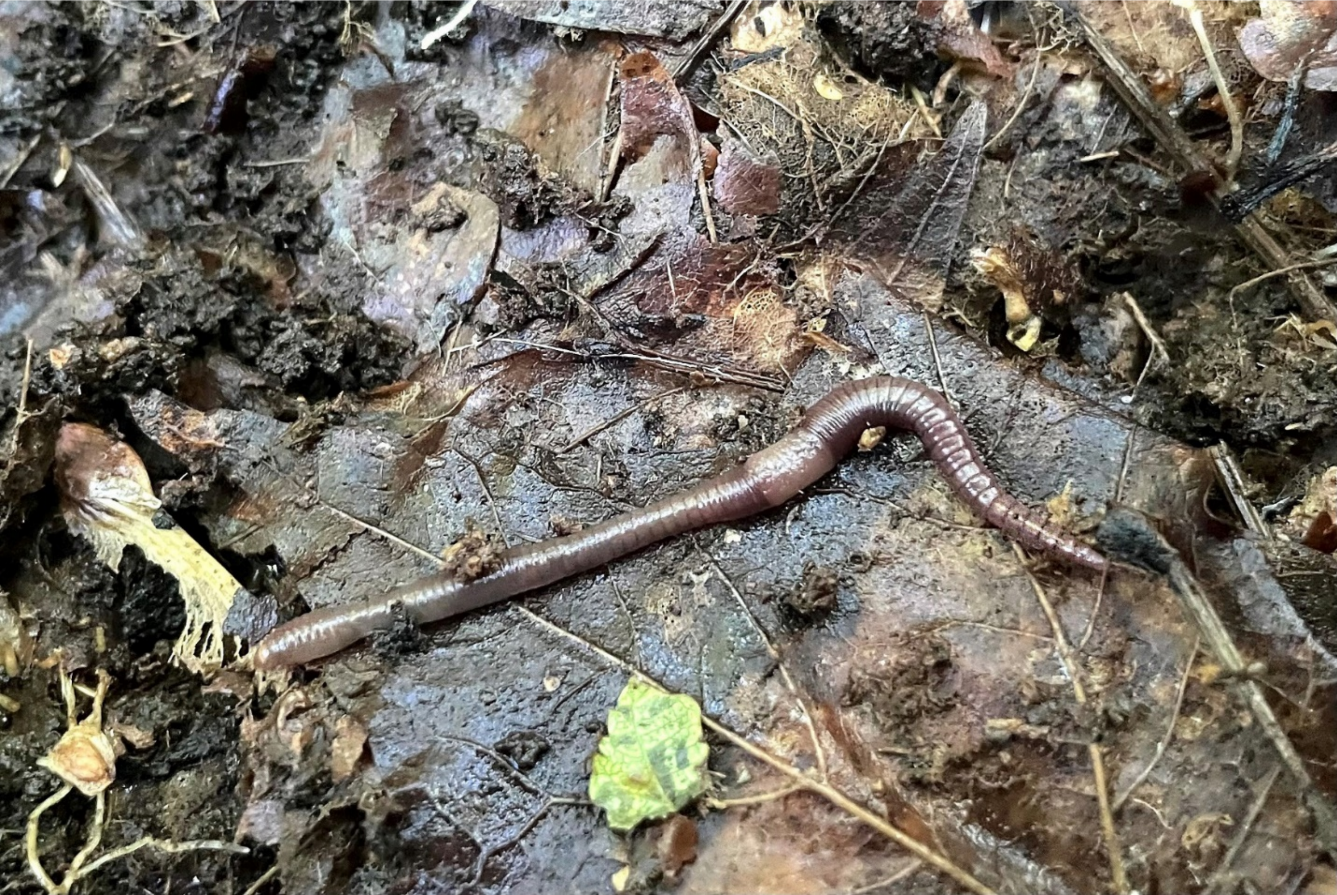
Adult *Bimastos rubidus* exposed underneath a moose dung pat in a hardwood stand on 19 August 2022 in the Kennedy Lakes Protected Natural Area, New Brunswick. *B. rubidus* lives mostly in leaf litter and under bark (Photo credit: Donald F. McAlpine/New Brunswick Museum).

The moose (*Alces alces*) has a wide distribution across forested boreal habitats in North America (Karns, 1998) and has apparently been present in New Brunswick (Canada) for some 2500 years (Boer, 1992). During the fall and winter, moose eat woody browse low in moisture, and fecal pellets are produced. However, in spring and summer, foods eaten are high in nutrients and water, and scat produced during spring and summer is soft and resembles that of domestic cattle (Schwartz & Renecker, 1998).

In pastureland, earthworms are known to aggregate under the dung pats of cattle (Holter, 1983). Bacher et al. (2018) found that four times as many earthworms were present under cattle dung pats than in surrounding soil, and that recruitment occurred from an area of about 3.8 m^2^ surrounding each dung pat. Epigeic earthworm species in particular were attracted to dung pats. This is largely because earthworm populations are often food‐limited (Curry, 1998) and dung pats provide a concentrated, if ephemeral, food source. Although there exists a substantial literature dealing with cattle dung pats and earthworms (e.g., Bacher et al., 2018; Holter, 1983), studies of the relationships between native ungulates and earthworms in northern temperate forests are recent (Cope & Burns, 2019; Dávalos, Nuzzo, et al., 2015a; Dávalos, Nuzzo, et al., 2015b; Dávalos, Simpson, et al., 2015; Dobson et al., 2023; Reed et al., 2023) Furthermore, all of these previous studies have focused on white‐tailed deer (*Odocoileus virginianus*) and non‐native earthworms deemed invasive. Here we document an apparent association between North American moose and the native earthworm, *B. rubidus*.

From 16 to 20 August 2022, spring–summer moose dung pats were located by systematically searching a hardwood stand in the Kennedy Lakes Protected Natural Area in north‐central New Brunswick. The stand occupied about 0.5 km^2^ centered on 46.822419° N, 66.626731° W. The site was dominated by sugar maple (*Acer saccharum*) and yellow birch (*Betula alleghaniensis*) with an open understory of lady fern (*Athyrium filix‐femina*) and (well browsed) hobble bush (*Viburnum lantanoides*). At each dung pat discovered (*N* = 21) a 0.25 m^2^ quadrat square was placed on the forest floor encompassing the pat. Dung pats were often colonized by the ascomycete fungus *Cheilymenia magnipila*, and pats had presumably been present for some months. A control square lacking a pat was established immediately adjacent to each quadrat containing a pat (Figure 2). We bagged each moose pat for later examination in the lab. Soils (podzols) at the site are moderately well‐drained, non‐compacted silt‐loam tills, with a coarse fragment content of <20%–50% (percentage of soil particles >2 mm) overlying metasedimentary and igneous parent materials (Colpitts et al., 1995). In the field, the upper organic (O horizon) soil layer was removed from each quadrat down to the top of the soil surface (A horizon), placed in a plastic tote, and with the aid of a headlamp, systematically searched for earthworms. The A horizon (10–15 cm) was then removed down to a gray mineral soil (E horizon) overlying granitic boulders and likewise searched for earthworms. All earthworms encountered were retained. Finally, bagged moose pats were turned into plastic totes in the lab, weighed, and all earthworms collected. All earthworms discovered, whether in the field or the lab, were killed in 70% ETOH, fixed in 10% formalin, and later stored in 70% ETOH. Worms were identified to species using Reynolds (2022) and assigned to one of three age classes: juvenile, aclitellate adults, or clitellate adults. To accommodate the relatively large number of control quadrats represented by zeros (no earthworms present–45%), we used a Wilcoxon rank‐sum test for two group comparisons with excessive zeros (Wang et al., 2023) to test for differences in *B. rubidus* numbers between quadrats encompassing moose dung and those lacking dung (*N* = 20 pairs) that supported *B. rubidus*. We used a standard Wilcoxon rank‐sum test to examine differences between control quadrats and those few with dung that included *Apporrectodea turgida* (*N* = 3 pairs) or *Lumbricus rubellus* (*N* = 3 pairs). All tests were run in R (R Core Team, 2020).

**FIGURE 2:**
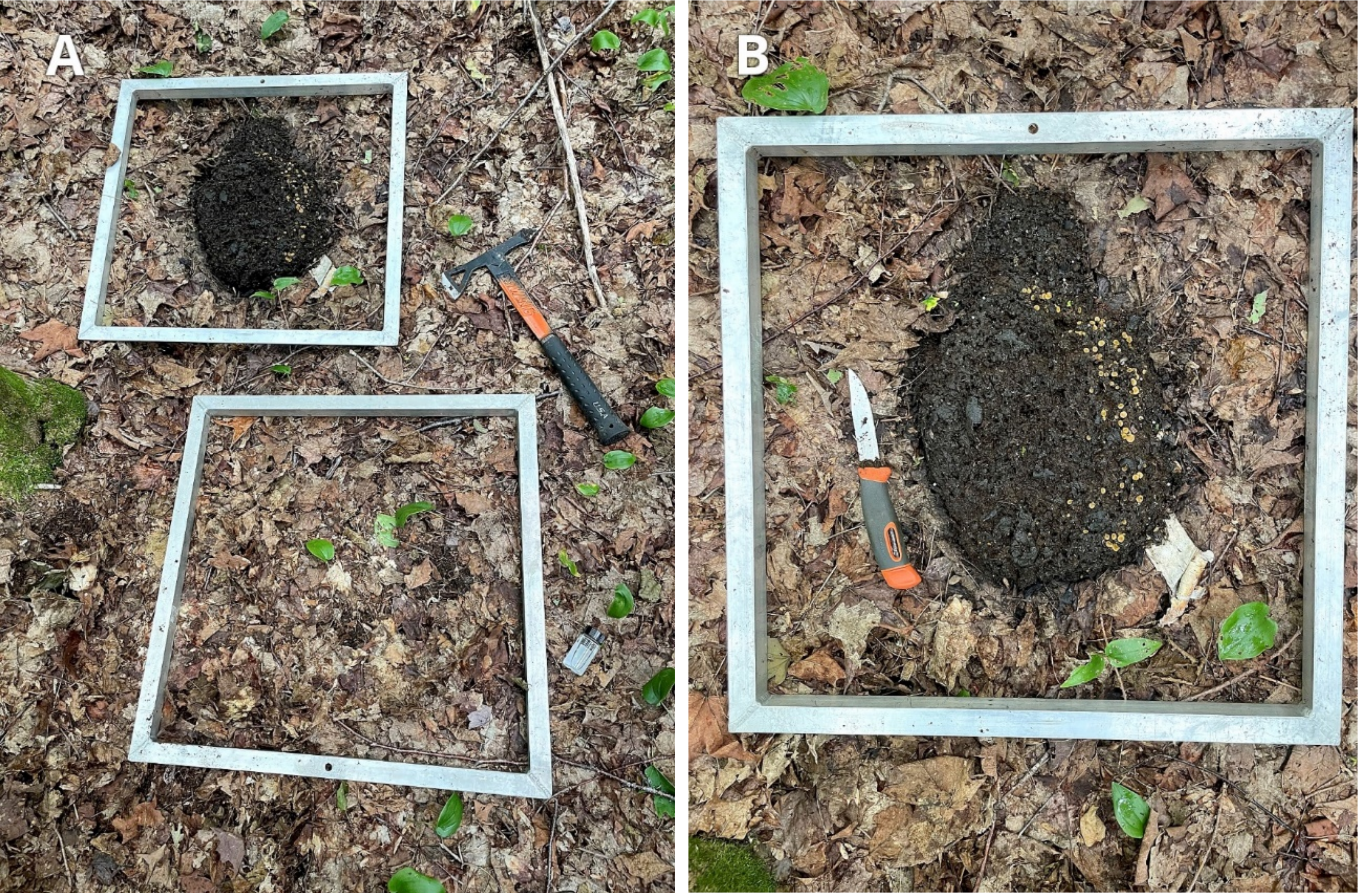
(A) Quadrat of 0.25 m^2^ encompassing a spring–summer moose dung pat, with adjacent control quadrat lacking dung, on the floor of a hardwood stand in the Kennedy Lakes Protected Natural Area, New Brunswick, 17 August 2022. (B) Dung pats were often colonized by the ascomycete fungus *Cheilymenia magnipila* (Photo credit: Donald F. McAlpine/New Brunswick Museum).

Table 1 reports the total number of earthworms recorded from the 21 paired 0.25 m^2^ quadrats containing moose dung and adjacent quadrats lacking dung (controls), as well as the range and mean number of earthworms per species per square meter. Wet weights for dung pats ranged from 197.4 to 1798.7 g ($\bar{x}=891.1\;g$, *N* = 21). There was no correlation between earthworm numbers/pat and dung weight (*r* = −0.0927, *p* = 0.6997, *p* < 0.05). Three species of earthworms were recorded in control quadrats; *B. rubidus* and small numbers of the non‐native lumbricids *A. turgida* and *L. rubellus*. Quadrats with dung supported four species; *B. rubidus* and likewise a smaller, but somewhat higher, numbers of the European lumbricids; *A. turgida*, *Dendrobaena octaedra*, and *L. rubellus*. Overall there were 7.4 times as many European lumbricids associated with moose dung than quadrats lacking dung. However, most of this difference can be ascribed to a single European species—*L. rubellus*. In paired comparisons, there was no significant difference in the total number of earthworms for the two European species associated with both controls and quadrats with dung, although sample sizes were small (*A. turgida*: *p* = 1.0, *W* = 4.5, df = 1, *p* < 0.1, *N* = 3; *L. rubellus*: *p* = 0.1, *W* = 0, df = 1, *p* < 0.1, *N* = 3). In contrast, there were 30.3 times as many *B. rubidus* collected from dung quadrats than those lacking dung, a highly significant difference (*p* = 0.0000339, *W* = 4.15, df = 1, *p* < 0.01). Maximum number of *B. rubidus* per square meter for control quadrats was 44 m^−2^ versus 872 m^−2^ for those quadrats with moose dung. Also noteworthy, age class data reveals that 80.0% of *B. rubidus* in dung quadrats were juveniles. Only 3 of the 20 quadrats with moose dung lacked juveniles. One contained no earthworms at all, while the other two each supported only a single adult *B. rubidus*. Conversely, only 44.1% of *B. rubidus* in controls were juveniles (Table 1), with five controls lacking any juveniles at all.

**TABLE 1:** Total number of lumbricid earthworms (±SD) collected from paired 0.25 m^−2^ quadrats with Moose (*Alces alces*) dung pats present and without (control).

	Moose dung				Control—No dung		
	*Apporectodea turgida*	*Bimastos rubidus*	*Dendrobaena octaedra*	*Lumbricus rubellus*	*Apporectodea turgida*	*Bimastos rubidus*	*Lumbricus rubellus*
Total no. earthworms collected^a^	2 (2‐0‐0)	1030 (824‐56‐150)	14 (10‐0‐4)	87 (64‐5‐18)	4 (0‐0‐4)	34 (15‐4‐15)	0 (6‐1‐3)
No. quadrats containing earthworms	3	20	1	3	3	20	3
Range in no. earthworms/m^2^	0–8	0–872	56	0–148	0–16	0–44	0–32
Mean no. earthworms/m^2^	0.8 ± 2.1	205.8 ± 270.6	…	116 ± 42.5	0.8 ± 2.1	7.0 ± 10.7	13.3 ± 13.6

^a^ Across 21 paired 0.25 m^2^ quadrats (by age class). Total number per quadrat is followed by an age classification formula, with the series of numbers separated by dashes indicating the number of juvenile–aclitellate, adult–clitellate, adult earthworms.

We have shown that spring–summer moose dung pats appear to provide important nursery sites for *B. rubidus* in hardwood stands. Because *B. rubidus* is largely a surface dwelling inhabitant of downed woody debris and leaf litter, this species may also be at greater risk of desiccation than deeper burrowing (anecic) earthworm species. Moose pats may therefore provide a refuge during periods of dry weather for this epigeic earthworm. Moose pats also clearly provide islands of elevated nutritional opportunity for *B. rubidus*.

Earthworms play significant roles in forest and soil ecology and can affect soil chemistry, vegetation, and wildlife (Brown et al., 2004). The impact of earthworms can be so profound they have been referred to as “ecosystem engineers” (Le Bayon et al., 2017). Studies of invasive earthworm‐white‐tailed deer interactions in temperate forests have documented outcomes that include increased earthworm densities associated with deer, that smaller earthworms show higher biomass in the presence of deer, and that populations of deer and earthworms interact to influence understory plants (Cope & Burns, 2019; Dobson et al., 2023; Reed et al., 2023). However, what role *B. rubidus*, or *B. rubidus* together with moose, might play in forests within their native range is unclear. While much attention has focused on the negative effects of non‐native earthworms in North America (Chang et al., 2021; Shartell et al., 2012), by contrast, little information exists on interactions between wildlife and the eight earthworm species native to Canada. Laakso and Setälä (1997) report a possible mutualistic relationship between *B. rubidus* (as *D. rubidus*) and the red wood ant (*Formica aquilonia*) in Finland in which earthworm biomass was seven times greater in ant nest mounds than in surrounding forest soils.

Although earthworms may act as vectors for the transmission of larval parasites, they may also be an important epidemiological element in reducing the number of infective larvae. Grønvold (1987) demonstrated that where earthworm activity led to the disintegration of cow pats in Denmark, there was a 50% reduction in the presence of infective trichostrongyle larvae adjacent to pats. d'Alexis et al. (2009) also found a significant reduction in the presence of parasitic larval nematodes in goat feces in the presence of a native earthworm in an experimental setting in Guadeloupe, West Indies. Moose are subject to a variety of endoparasites, although many of these do not appear to cause gross pathology (Lancaster & Samuel, 1998). The notable exception is the nematode *Parelaphostrongylus tenuis*. Whether *B. rubidus* has any influence on the prevalence of parasites in moose is unknown.

Newton (2018) notes that cow pats in Britain provide a concentrated food source of invertebrates, including earthworms, for many bird species. Waite (1981) and Briggs (1984) report that Rooks (*Corvus frugilegus*) and Oystercatchers (*Haematopus ostralegus*) respectively, exploit the concentrations of earthworms in and under cow pats. Whether moose pats are exploited seasonally as a source of earthworm prey by North American forest birds is unknown. Reynolds (1977) reported *B. rubidus* among the stomach contents of American Woodcock (*Scolopax minor*), but otherwise, little is known about the natural predators of *B. rubidus* in the species native habitat.

In conclusion, the presence of spring–summer moose pats would seem to enhance populations of the native earthworm *B. rubidus*. Whether elevated *B. rubidus* populations, made possible by the presence of moose, have any influence on local forest ecology remains to be determined.

## CONFLICT OF INTEREST STATEMENT

The authors declare no conflicts of interest.

## Data Availability

Earthworm and fungal vouchers and associated data have been deposited in the New Brunswick Museum collections and are accessible at https://biodiverse-nb.ca/portal/index.php by searching taxa names in conjunction with the query “Kennedy Lakes Protected Natural Area.”
